# Evaluation of Neutrophil Elastase Inhibitors as Potential Therapies for *ELANE* Associated Neutropenia

**DOI:** 10.33696/immunology.6.208

**Published:** 2024

**Authors:** Vahagn Makaryan, Merideth Kelley, Audrey Anna Bolyard, Gobind Chugh, David C. Dale

**Affiliations:** 1 Department of Medicine, University of Washington, Seattle, Washington, U.S.A.

**Keywords:** Neutropenia, ELANE, Neutrophil elastase, Congenital neutropenia, Cyclic neutropenia, Neutropenia therapy, MK0339

## Abstract

Neutrophil elastase (*ELANE*) mutations are the most common cause of cyclic (CyN) and congenital neutropenia (SCN), two autosomal dominant disorders causing recurrent infections due to impaired neutrophil production. Granulocyte colony-stimulating factor (G-CSF) corrects neutropenia but has adverse effects, including bone pain and in some cases, an increased risk of myelodysplasia (MDS) and acute myeloid leukemia (AML). Hematopoietic stem cell transplantation is an alternative but is limited by its complications and donor availability. Alternative therapies are needed, particularly for patients with poor responses to G-CSF and those at higher risk of MDS/AML.

We previously reported that cell-permeable neutrophil elastase (NE) inhibitors are a potential treatment for *ELANE* neutropenia, based on studies using HL-60 cells. Our hypothesis was that mutant NE was not properly stored to the neutrophil granules and thereby caused cytoplasmic damage, activation of apoptotic pathways and neutropenia. We have extended this work using CD34^+^ cells from patients with *ELANE* mutations and several selective NE inhibitors, i.e., MK0339, sivelestat, BAY-678, and GW311616, as well as the DDP1 inhibitor, brensocatib.

Only MK0339 restored neutrophil differentiation with an increase in the proportion of neutrophil marker-positive cells (CD66b^+^/CD14^+^ and CD11b^+^/CD15^+^). In contrast, other NE inhibitors, i.e., sivelestat, BAY-678, and GW311616 and the DPP1 inhibitor, brensocatib, showed no effect on neutrophil differentiation. Molecular docking studies showed that MK0339 binds to an alternative site on the NE protein compared to other inhibitors with greater inhibitor-NE protein stability, suggesting a unique mechanism of action and supporting further investigation of MK0339 as a therapy for *ELANE* associated neutropenia.

## Introduction

Mutations in the gene for neutrophil elastase (*ELANE*) are the most common cause for cyclic (CyN) and congenital neutropenia (SCN) [1–3]. The majority of patients with these autosomal dominant disorders can be effectively treated with the granulocyte colony-stimulating factor (G-CSF) [4,5]. G-CSF is administered subcutaneously, daily, every other day or several times per week and it has expected adverse effects. These are most commonly bone pain, headache and arthralgias, but for some patients it may predispose to the development of myelodysplasia and or acute myeloid leukemia [6,7]. Although this risk is difficult to quantitate, the concern leads to hesitancy by patients, families and physicians with long-term administration, particularly at high doses [8]. The only other effective treatment currently is hematopoietic stem cell transplantation, and therapy is limited by availability of a suitable donor, the inherent risk of graft versus host disease and the cost and complexity of this treatment [9–11]. Numerous other therapies have been tried without consistent benefit [12].

*ELANE*, located on chromosome 19, encodes a 29 kd powerful protease which has many potential substrates, including G-CSF [13]. Mutations in this protease were first recognized in CyN and then in SCN because of the overlapping presentations and symptoms for these conditions, largely based on family studies [14]. With availability and wide use of Sanger and exome sequencing, more than 200 different mutations have been identified [1,15]. The clinical phenotypes vary from mild to severe, the more severely affected patients respond poorly to G-CSF and are at greatest risk of evolution to MDS and AML. The mutations G214R, C151Y, and C233X portend the greatest risk [15]. Because the risk of this evolution cannot be clearly separated from G-CSF treatment, we and other investigators have sought alternative therapies.

We have pursued the possibility that inhibitors of neutrophil elastase might be alternatives to G-CSF over several years [16]. We began these investigations based on the hypothesis that the mutant enzyme was the sole cause for these diseases. We found that one inhibitor called MK0339 was consistently the best of the tested inhibitors to promote cell survival and differentiation of HL-60 cells expressing mutant *ELANE* [17]. Recently we have solidified this hypothesis through gene knockout and gene editing studies [18,19]. We have now expanded on this work comparing several selective inhibitors of NE using CD34^+^ cells from patients with SCN and CyN. We observed that only some of the cell permeable NE inhibitors permit differentiation; others are inactive across a spectrum of CD34^+^ cells from patients. In this report we provide structural modeling data on NE and its interactions with potential therapeutic NE inhibitors to enhance these observations. We believe this work is foundational for advancing the possibility of a novel oral therapy for *ELANE* associated neutropenia.

## Methods and Materials

### IRB approval

The Institutional Review Board of the University of Washington approved these studies. Informed written consent was obtained from all of the subjects. Bone marrow samples were collected in association with an annual follow-up as recommended by the Severe Chronic Neutropenia International Registry.

### Patients’ cells: Bone marrow: Separation, CD34 cell preservation and expansion

Freshly collected, anticoagulated bone marrow (3–6 ml) was shipped overnight at an ambient temperature. Hematopoietic stem and progenitor cells (HSPC’s) were initially enriched using RosetteSep Human Bone Marrow Progenitor Cell Pre-Enrichment Cocktail, (Cat. no.15027; All catalog numbers refer to materials from StemCell Technologies unless indicated otherwise) and Lymphoprep (Cat.no. 07801) according to manufacturer’s protocol. The HSC enriched cell population was expanded by culturing for 4 days in CD34^+^ expansion media (StemSpan SFEMII media (Cat.no. 09655) supplemented with 1% Penn Strep (Cat.no 03–031-1B, Biological Industries), 1x StemSpan CD34^+^ Expansion Supplement (10x) (Cat.no. 02691), and 1.0 μM UM729 (Cat.no.72332), at 37°C 5% CO_2_. After expansion, CD34^+^ cells were further enriched using EasySep Human CD34 Positive Selection Kit II (Cat.no. 17856) according to manufacturer’s protocol. Cell counts and viability were evaluated using an Invitrogen Countess and trypan blue. Enriched CD34^+^ cells were cryopreserved at 400×10^5^ cells/ml in Cryostor CS10 (Cat.no. 07931). Cells were stored in liquid nitrogen, vapor phase.

### Leukapheresis cells

CD34^+^ cells were collected following 5 days of subcutaneous G-CSF administration (10 mcg/kg/day). On day 5 plerixafor was administered once subcutaneously (240 mcg/kg/dose) at Bloodworks Northwest, Seattle WA and peripheral blood stem cells (PBSC) collected by standard institutional procedures. CD34^+^ cell enrichment was performed on the freshly mobilized PBSCs using CliniMACS and cryopreserved following institutional Standard Operating Procedure. For controls, cryopreserved healthy human CD34^+^ progenitor cells from mobilized peripheral blood were obtained from Lonza (Cat no. 4Y-101C).

### Differentiation assay

Previously frozen CD34^+^ cells thawed and were allowed to recover for 3 days in CD34^+^ expansion media and were subjected to a differentiation protocol adopted from Nasri *et al.* [20]. In brief, HSCs were cultured for 7 days in RPMI (Cat. no 11875093, Gibco^™^) supplemented with 1% Glutamax (Cat.no 35050061, Gibco^™^), 10% FBS (Cat.no 04–001-1A, Biological Industries), 5 ng/ml IL-3 (Cat.no 200–03), SCF (Cat. no 300–07), GM-CSF (Cat.no 300–03) & 10 ng/ml G-CSF (Cat. no 300–23), all from PeproTech, for proliferation and myeloid progenitor differentiation followed by a 7-day culture in RPMI, 1% Glutamax, 10% FBS, 1% Penn Strep (Cat.no 03–031-1B, Biological Industries), 10 ng/ml G-CSF for neutrophil differentiation and maturation. Cell counts and viability were evaluated using an Orflo Moxi V and propidium iodide.

### Cytospin staining

8 × 10^4^ cells at day 15 of differentiation were spun onto Cytoslide microscope slides (ThermoFisher) using Cytospin 4 low speed cytocentrifuge (Thermo Scientific) and stained with Kwik-Diff staining system (MilliporeSigma, eosin/methylene blue) according to manufacturer’s recommendations. Microphotographs were taken on LEITZ LABORLUX S polarizing light microscope at 400X magnification using Nikon DSLR digital camera.

### Flow cytometry

Myeloid maturation of CD34^+^ cells was analyzed at day 14 of the differentiation by flow cytometry utilizing antibodies characterizing the neutrophilic lineage. Pacific Blue-CD66b anti-human (Cat no. 305112, Biolegend), APC-CD14 anti-human (Cat no. 130–110-520, Miltenyi Biotec), APC-CD11b anti-human (Cat no. 130–110-554, Miltenyi Biotec) and Pacific Blue-CD15 anti-human (Cat no. 130–113-488, Miltenyi Biotec) were used. Cell debris was gated out by using a zombie yellow viability kit (Cat no. 423103, Biolegend). Doublet discrimination gating was used for exclusion of doublets. Appropriate isotype controls were used to help distinguish between specific and non-specific antibody bindings. For analyzing cell population of interest, quadrant gate analysis method was used for double positive cells selection.

### Molecular modelling

A computational analysis of the neutrophil elastase (NE) protein was conducted using the Molecular Operating Environment 2024 (MOE) software [21]. In this study, Mol2 files of the inhibitors and the PDB file of the wild-type NE, obtained from UniProt, were utilized. MOE was employed to simulate the docking of various inhibitors at specific binding sites on the NE protein. These binding sites were computationally selected based on several factors, including site size, steric hindrance, electrostatic repulsion, and amino acid compatibility. For each inhibitor, 50 docking trials were performed in triplicate, resulting in a total of 150 docking attempts per inhibitor across the four tested inhibitors. The stability of each protein-ligand complex was assessed using the free energy of binding (S score in kcal/mol), providing an approximation of binding stability. The accuracy of this S score was also given as a root mean square deviation (RMSD), with scores <2.0 indicating good conformation, scores 2.0–3.0 indicating acceptable conformation, and >3.0 indicating poor conformation.

### Neutrophil elastase proteolytic activity inhibition

The NE proteolytic activity in cell lysates from day 14 differentiated CD34^+^ cells exposed and not exposed to the inhibitors was determined using EnzCheck Elastase assay kit from Thermo Fisher Scientific, according to the manufacturer’s recommendations.

### Inhibitors

MK0339 NE inhibitor was provided by Merck & Co. (Kenilworth, NJ, USA). The same inhibitor is also commercially available through MedChemExpress (MCE) as DMP-777 (HY-75957). NE inhibitors: Sivelestat (HY-17443), GW311616 (HY-15891), BAY-678 (HY-111457A) with its inactive control, BAY-677 (HY-111457) and DPP1 inhibitor Brensocatib (HY-101056) were purchased from MedChemExpress (Monmouth Junction, NJ).

## Results

### Patients and *ELANE* mutations

5 patients previously enrolled in the Severe Congenital Neutropenia International Registry (SCNIR) participated in the study (Table 1).

### Inhibitors, concentrations, proteolytic activity inhibition and toxicity studies

All the inhibitors used in this study are small molecule cell-penetrant chemicals with well characterized chemical and biological properties and have been used or investigated by other groups (Table 2).

**Figure 1.**
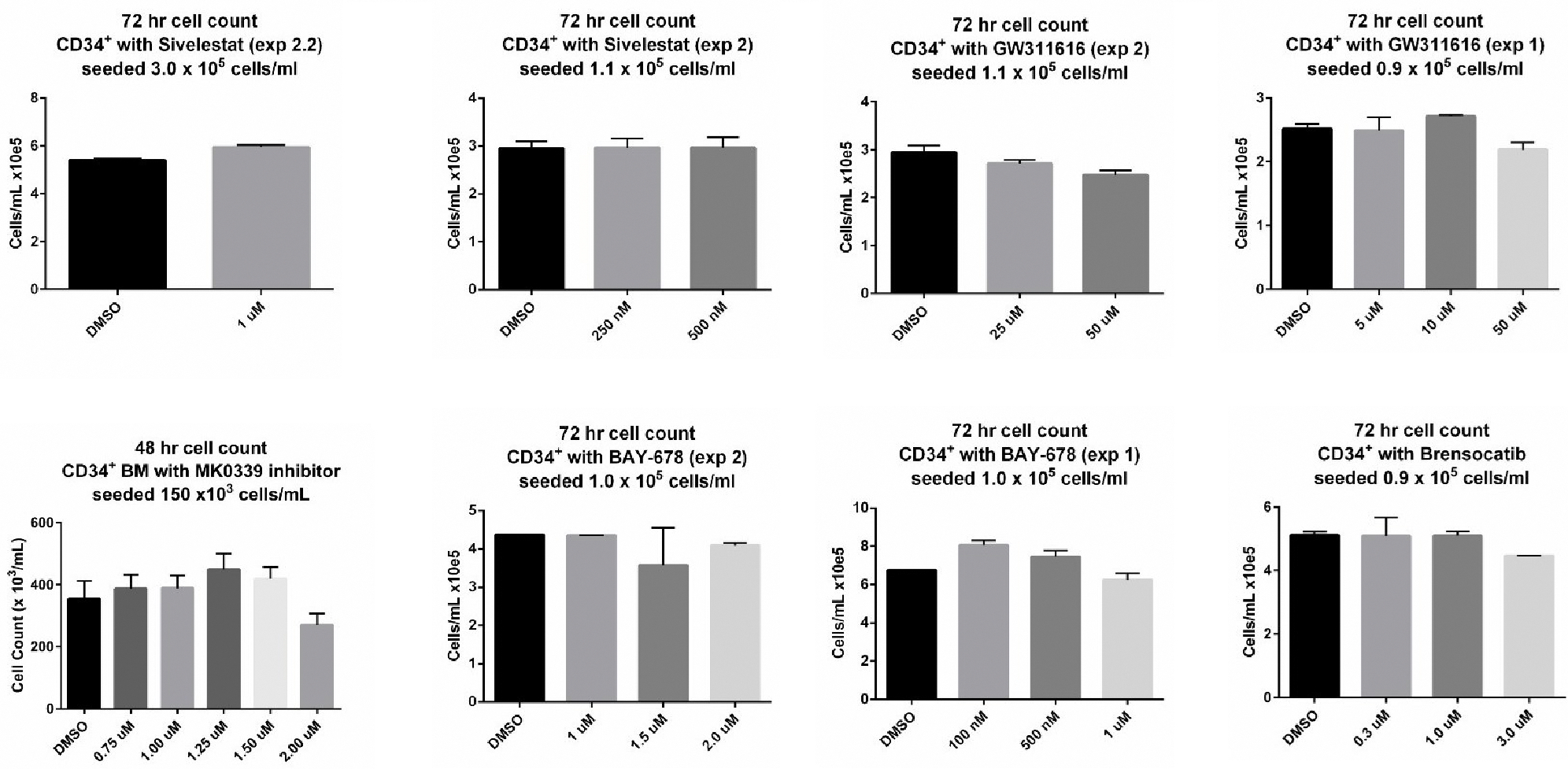
The effect of NE inhibitors (MK0339, BAY-678, sivelestat, GW311616) and the DPP1 inhibitor brensocatib on the proliferative capacities of CD34^+^ cells. CD34^+^ cells were cultured with the inhibitors at the concentrations indicated in the graphs for 48 and 72 hours (Mean cell counts with standard deviation).

We investigated the potential toxic effects of these compounds on CD34^+^ cells in dose-response studies. Effects on cell counts and viability of CD34^+^ cells were examined in 48 and 72 h culture experiments at 37°C in a standard CO_2_ incubator (Figure 1). Based on the results of these studies, subsequent experiments were performed with inhibitors at a concentration of 1.25 uM for MK0339, 1 uM for Sivelestat, BAY-678, BAY-677, and brensocatib and 10uM for GW311616 unless otherwise specified. It is important to note, that in addition to 4 NE inhibitors we have also used brensocatib, a dipeptidyl peptidase 1 (DPP1) inhibitor, which also inhibits NE but indirectly, through inhibiting DPP1 which is an important factor for NE activation.

### NE inhibition

**Figure 2.**
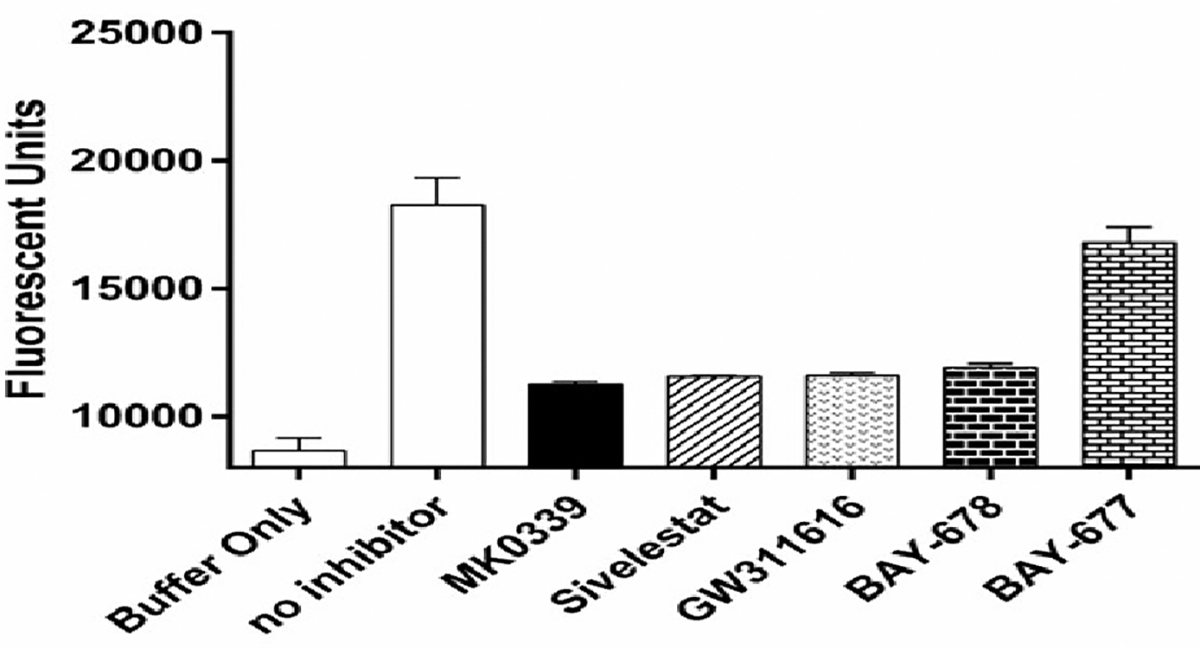
The effect of NE inhibitors on proteolytic activity of NE. CD34^+^ cells derived from healthy volunteers were differentiated for 14 days and subsequently lysed. The lysates were incubated with or without inhibitors, following the manufacturer's protocol. BAY-677, the inactive analog of inhibitor BAY-678, was included as a negative control. Neutrophil elastase proteolytic activity was measured using the EnzChek elastase assay kit and quantified with a fluorescence microplate reader (Mean Fluorescence Units with standard deviation).

We measured NE proteolytic activity and its inhibition with addition of NE inhibitors in the cell lysate of differentiated granulocytes at day 14 of differentiation by utilizing EnzCheck Elastase assay kit from Thermo Fisher Scientific. We determined that all 4 NE inhibitors have similar performance and are blocking the enzyme’s proteolytic activity by 35% to 38% across the line at the concentrations used in the cell culture in this study (Figure 2).

### Myeloid differentiation

**Figure 3.**
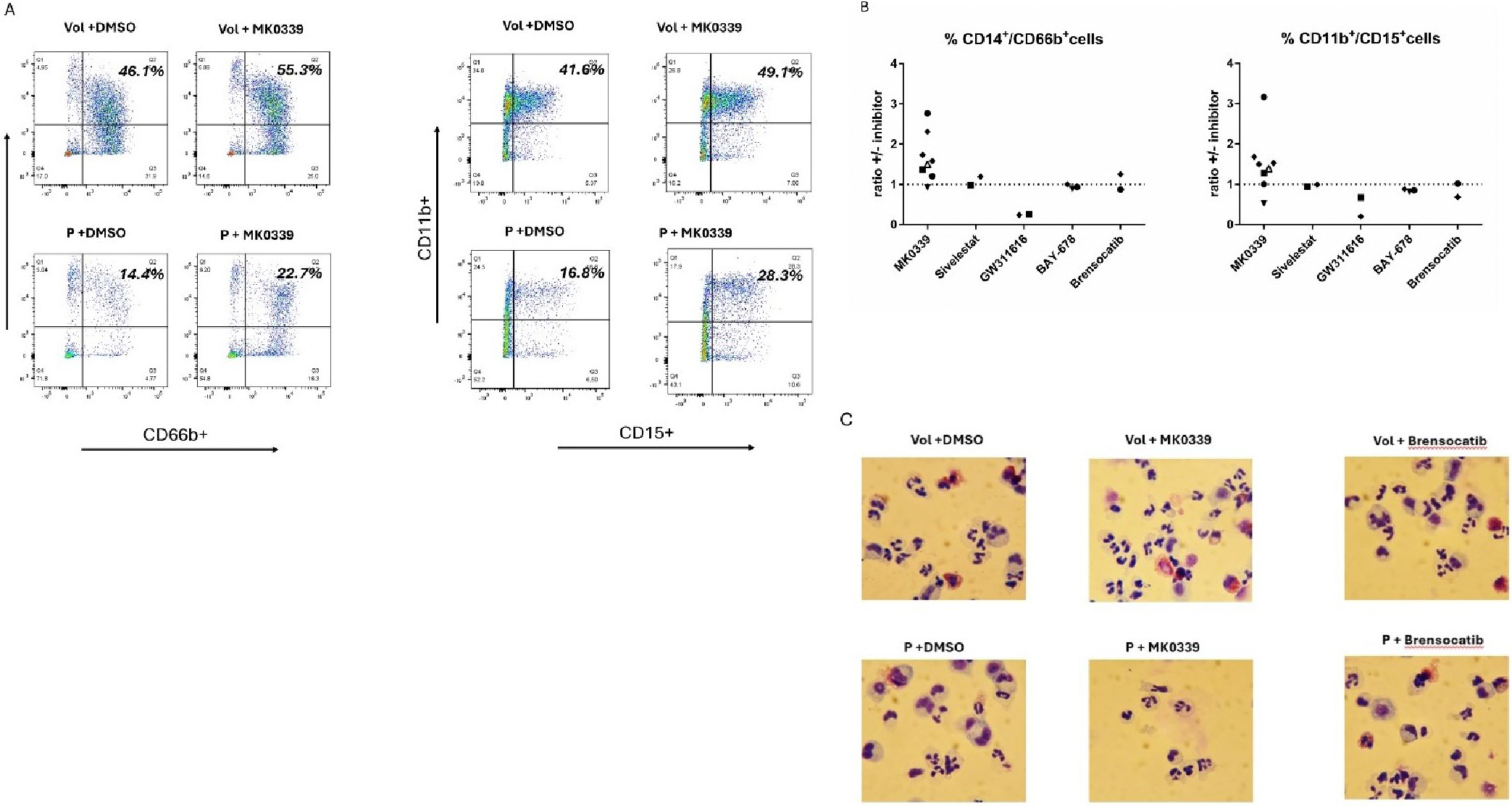
The effect of NE inhibitors (MK0339, BAY-678, sivelestat, GW311616) and the DPP1 inhibitor brensocatib on the myeloid differentiation and maturation of CD34^+^ cells. Healthy volunteer and patient derived CD34+ cells were differentiated for 14 days in the presence or absence of inhibitors. The resultant cells were labeled with antibodies to CD14, CD66b, CD11b, and CD15 surface markers and analyzed using flow cytometry. **A.** Representative experiment histograms are shown. The proportion of CD14^+^/CD66b^+^ and CD11b^+^/CD15^+^ positive cells in quadrant 2 are indicated. **B.** Graphical representation of the percentage of CD66b^+^/CD14^+^ and CD11b^+^/CD15^+^ cellular subsets of the patients' cells after the addition of NE inhibitors. For each individual experiment, the percentage of cells with a mature phenotype after addition of inhibitor was divided by the percentage measured when only the vehicle control was added and this ratio was plotted. Data from 5 different patients, represented in at least two different experiments. Each individual patient has a different symbol. **C.** Cell cytospins stained with Kwik-Diff (eosin/methylene blue) were imaged using a Nikon digital camera. Cell differentiation was evaluated at 400x magnification by light microscope. Representative experiments showing the effect of MK0339 and brensocatib inhibitors on healthy volunteer and patient cells are shown.

We obtained about 2 million cells with at least 95% viability from bone marrow samples of 5 different patients with *ELANE* neutropenia, as well as from healthy volunteers. Purified CD34^+^ cells were pushed towards myeloid differentiation in the presence and absence of MK0339, sivelestat, BAY-678 and GW311616 NE inhibitors and cultured as described above. On day 14, cells were labeled with CD66b/CD14 and CD11b/CD15 myeloid differentiation surface markers and assessed by flow cytometry. The proportion of CD66b^+^/CD14^+^ and CD11b^+^/CD15^+^ cells was 2-to-3-fold lower in all 4 patient cell lines compared to the volunteers (data not shown), showing impairment of myeloid differentiation consistent with our previous report [19]. Addition of sivelestat, BAY-678 and GW311616 NE and brensocatib inhibitors did not reveal any positive effect on the patient derived cell lines. The addition of MK0339 exhibited a restoration effect on the impaired cell differentiation in the cells from 4 of the 5 patients with an increase of CD66b^+^/CD14^+^ and CD11b^+^/CD15^+^ subsets to up to 3-fold (Figure 3A and 3B). Inhibitor treatment of volunteer cells produced no noticeable differences in the proportions expressing these surface markers. Interestingly, GW311616 had a negative effect on both volunteer and patient derived cells myeloid differentiation; it decreased the proportion of cells of both CD66b^+^/CD14^+^ and CD11b^+^/CD15^+^ subsets.

Microscopic examination utilizing Kwik-Diff staining of the differentiated CD34^+^ cells revealed a typical block of differentiation in the promyelocytic stage of all patient derived cell lines compared to the volunteers, which was partially resorted by the addition of MK0339 inhibitor. There were no effects from sivelestat, BAY-678 and GW311616 NE and brensocatib inhibitors (Figure 3C).

### Molecular modelling and NE inhibitors docking analysis

To gain deeper insight into the interaction between the inhibitor and NE protein, including their binding specifics, we conducted molecular modeling using the MOE software platform. Molecular docking analysis of 4 small molecule, cell permeable NE inhibitors (MK0339, sivelestat, GW311616, BAY-678) and inactive control, BAY-677, revealed an alternative binding site for MK0339 inhibitor compared to all others.

**Figure 4.**
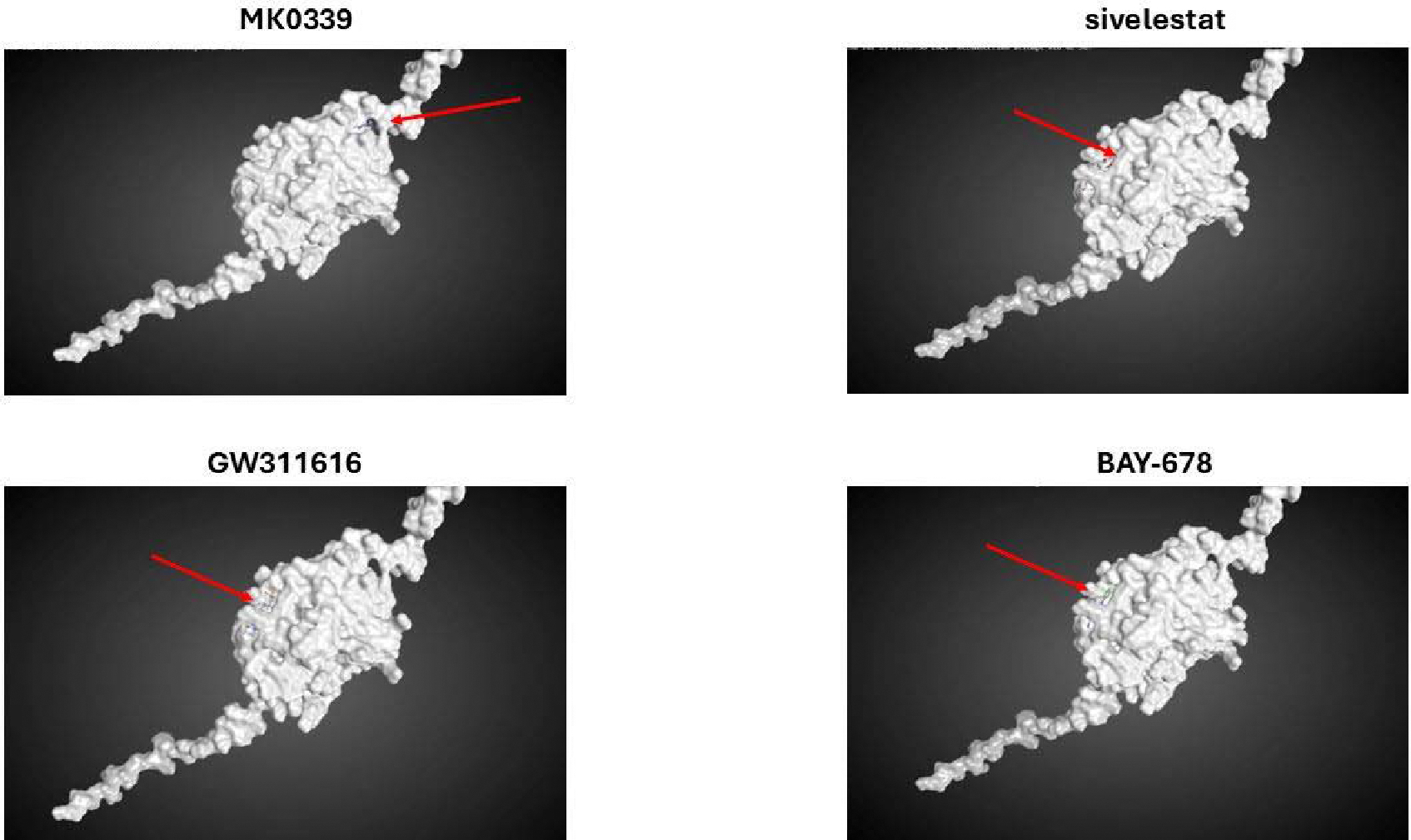
Molecular docking simulation analysis. Molecular docking simulations for NE inhibitors were conducted using the MOE software package. The simulations utilized *ELANE* G214R, P139L, and wild-type models of neutrophil elastase. The images captured from the simulations display the binding sites of the inhibitors MK0339, sivelestat, GW311616, and BAY-678 with the wild-type NE. The inhibitor molecules are highlighted in color and indicated by arrows.

MOE software analysis showed that sivelestat, GW311616 and BAY-678 have similar binding sites with NE molecule. Sivelestat, BAY-678, and GW311616 inhibitors bind with amino acid chains consisting of amino acids 139–146 and 233–239 for sivelestat, 140–145 and 235–240 for BAY-678, and 139–146 and 233–239 for GW311616. Interestingly, MK0339 binds at the opposite side of the molecule. Compared to the other NE inhibitors used in this study, MK0339 binds to three distinct amino acid chains corresponding to amino acids at 250–255, 122–130, and 98–104 positions, suggesting a different mechanism of inhibition (Figure 4). The calculated stability score of MK0339 inhibitor with the protein complex was −7.76, −7.20, and −8.21 for mutations G214R, P139L, and w-type NE, respectively (RMSD values of 1.08, 2.30, and 2.60, respectively). Interestingly, sivelestat, GW311616, and BAY-678 had higher values that were very similar to BAY-677, the inactive control (stability values were −6.76, −6.57, and −6.75 for G214R, P139L, and w-type NE respectively) (RMSD values of 2.81, 2.68, and 1.74, respectively). Higher values indicate a less stable inhibitor-protein complex (Table 3).

## Discussion

Severe congenital neutropenia (SCN) is a rare clinical disorder characterized by extremely low blood neutrophil counts that compromise the acute inflammatory response leading to recurrent and sometimes fatal bacterial infections. Bone marrow examinations usually reveal a focused abnormality in myeloid development with otherwise preserved hematopoiesis. Most marrow examinations show "maturation arrest," with a relative abundance of early myeloid precursors, i.e., promyelocytes, but few cells of this lineage beyond the myelocyte stage of development. The majority of these patients have mutations in *ELANE*, the gene for NE. This potent protease is synthesized during the promyelocyte and early myelocyte stage in neutrophil development. Most research studies indicate that the generation of the mutant enzyme initiates apoptosis of the developing myeloid cells via the unfolded protein response at this mid-point in neutrophil development. Exactly how this occurs is not yet known. We also do not know why some mutations cause mild and others more severe disease, i.e., the wide spectrum in the genotype-phenotype relationships or why some patients have regular variation in blood neutrophil counts, i.e., high to very low, in a cyclic pattern. However, recent studies indicate that knocking out the mutant gene and some inhibitors of the proteolytic activity of NE can correct the cellular abnormality in laboratory models [18–20].

Results of this study provide encouraging data to support the development of NE inhibitors to treat *ELANE*-associated neutropenia. To extend our previous work, we examined the effects of several cell-permeable NE inhibitors on NE proteolytic activity and myeloid differentiation of CD34^+^ cells derived from patients with *ELANE* associated neutropenia, and performed molecular docking studies of the inhibitors with wild-type and mutant NE. We found that only one inhibitor, MK0339, restored neutrophil differentiation in patients’ cells and discovered unique properties which may account for this finding. More specifically, we found that the NE inhibitor MK0339 exhibits a unique mechanism of action compared to other inhibitors. This is demonstrated by its distinct binding site and its interaction with three amino acid chains, whereas other NE inhibitors typically bind to only two. The most important clinical finding was that MK0339 had positive effects on promoting myeloid differentiation in patient-derived CD34^+^ cells, similar to our previous report for MK0339 effects on HL60 cells expressing various *ELANE* mutations [18]. This result was observed across four out of five patient samples, with increase in the expression of key neutrophil markers, CD66b^+^/CD14^+^ and CD11b^+^/CD15^+^. In contrast, other inhibitors, i.e., sivelestat, GW311616, and BAY-678 had weaker binding and failed to improve differentiation, highlighting the specificity and potential therapeutic advantage of MK0339.

The molecular modeling and docking studies support a distinct activity of MK0339. Unlike the other NE inhibitors, which bind to the same site on the NE protein, MK0339 binds to an alternative site. This alternative binding could be responsible for the observed restoration of differentiation. The greater effectiveness of this inhibitor may also be attributed to a more stable inhibitor-protein complex as shown in the modeling studies for NE expressed by mutants G214R and P139L as well as with the w-type protein. Ultimately, higher stability scores associated with MK0339, compared to other inhibitors, may also influence pharmacokinetics and *in vivo* effectiveness. We were surprised to observe the negative effect of GW311616 on both patient and healthy donor-derived CD34^+^ cells. The reduction in the proportion of differentiated cells suggests that some inhibitors may have detrimental effects on neutrophil development.

Although the other NE inhibitors tested in this study (sivelestat, BAY-678, GW311616) were effective at inhibiting NE proteolytic activity, their lack of impact on myeloid differentiation raises questions about NE inhibition as a simple explanation for the effects that we observed. In support of this uncertainty, we observed that the DPP1 inhibitor brensocatib, which indirectly inhibits NE activation, also failed to demonstrate efficacy in restoring neutrophil differentiation.

This study represents an important step toward the development of alternative therapies for patients with *ELANE* mutations, particularly those who respond poorly to G-CSF and are at increased risk of progression to myelodysplastic syndrome (MDS) and acute myeloid leukemia (AML). However, there are substantial limitations to this study. The sample size was limited, and there is a great diversity of the mutations causing *ELANE* associated neutropenia. So, we cannot infer that all, or even most, patients’ cells will respond similarly. Additionally, while our molecular modeling provided valuable insights into inhibitor binding, further structural studies, including crystallography, could provide a more detailed understanding of how MK0339 interacts with mutant NE. Finally, *in vivo* studies will be necessary to determine the clinical efficacy and safety of MK0339 and any other NE inhibitors.

## Conclusion

In conclusion, our data support further investigations of MK0339 as a viable therapeutic candidate for *ELANE*-associated neutropenia. By restoring myeloid differentiation in patient-derived cells and exhibiting a stable binding profile with mutant NE, MK0339 presents a promising alternative to G-CSF.

## Figures and Tables

**Table 1.** Patients. Basic clinical information on the patients included in this study.

Patient #	Age	Gender	Diagnosis	ELANE mutation	G-CSF (Y/N)
**1**	25	M	SCN	G221X	Y
**2**	33	M	SCN	M154R	Y
**3**	43	M	SCN	P139L	Y
**4**	18	F	SCN	A57V	Y
**5**	21	M	SCN	A233P	Y

**Table 2.** Inhibitors. The manufacturers and working concentrations of the inhibitors used in this study are listed.

Inhibitor	Source	Concentration in culture
MK0339	Merck	1.25 uM
Sivelestat (ONO-5046)	MCE: HY-17443	1 uM
GW311616	MCE: HY-15891	10 uM
BAY-678	MCE: HY-111457A	1 uM
Brensocatib (AZD 7986)	MCE: HY-101056	1 uM
BAY-677 (inactive control)	MCE: HY-111457	1 uM

**Table 3.** Molecular docking simulation analysis. Molecular docking simulations for NE inhibitors were conducted using the MOE software package. The simulations utilized *ELANE* G214R, P139L, and wild-type models of neutrophil elastase. The stability of each inhibitor-protein complex was assessed using the free energy of binding (S score in kcal/mol), providing an approximation of binding stability.

Inhibitor	G214R	P139L	w-type NE
**MK0339**	−7.76	−7.20	−8.21
**GW311616**	−7.03	−6.78	−7.13
**sivelestat**	−6.68	−6.75	−6.68
**BAY-678**	−6.28	−5.79	−6.31
**BAY-677 (inactive)**	−6.76	−6.57	−6.75
